# Supplementary material for: Imiquimod Is Effective in Reducing Cervical Intraepithelial Neoplasia: A Systematic Review and Meta-Analysis
Source: Cancers (Basel). 2024 Apr 22;16(8):1610. doi: 10.3390/cancers16081610 (PMC11048968; doi:10.3390/cancers16081610)
Supplement: Supplementary file 1 [file cancers-16-01610-s001.zip › cancers-2954687-supplementary.pdf]

Supplementary Method

Search key

PubMed

(imiquimod OR aldara) AND (cervical neoplasia OR cervical cancer OR cervical dysplasia OR cervical intraepithelial neoplasia OR squamous intraepithelial neoplasia OR

CIN OR CIN1 OR CIN2 OR CIN3 OR HSIL OR LSIL OR ASCUS OR ASC-US OR ASC-H OR AGC OR atypical glandular cells OR AGC-NOS OR HPV OR human papillomavirus)

Embase

(imiquimod OR aldara) AND (cervical neoplasia OR cervical cancer OR cervical dysplasia OR cervical intraepithelial neoplasia OR squamous intraepithelial neoplasia OR

CIN OR CIN1 OR CIN2 OR CIN3 OR HSIL OR LSIL OR ASCUS OR ASC-US OR ASC-H OR AGC OR atypical glandular cells OR AGC-NOS OR HPV OR human papillomavirus)

CENTRAL

(imiquimod OR aldara) AND (cervical neoplasia OR cervical cancer OR cervical dysplasia OR cervical intraepithelial neoplasia OR squamous intraepithelial neoplasia OR

CIN OR CIN1 OR CIN2 OR CIN3 OR HSIL OR LSIL OR ASCUS OR ASC-US OR ASC-H OR AGC OR atypical glandular cells OR AGC-NOS OR HPV OR human papillomavirus)

Web of Science

(imiquimod OR aldara) AND (cervical neoplasia OR cervical cancer OR cervical dysplasia OR cervical intraepithelial neoplasia OR squamous intraepithelial neoplasia OR

CIN OR CIN1 OR CIN2 OR CIN3 OR HSIL OR LSIL OR ASCUS OR ASC-US OR ASC-H OR AGC OR atypical glandular cells OR AGC-NOS OR HPV OR human papillomavirus)

Scopus

(imiquimod OR aldara) AND (cervical neoplasia OR cervical cancer OR cervical dysplasia OR cervical intraepithelial neoplasia OR squamous intraepithelial neoplasia OR

CIN OR CIN1 OR CIN2 OR CIN3 OR HSIL OR LSIL OR ASCUS OR ASC-US OR ASC-H OR AGC OR atypical glandular cells OR AGC-NOS OR HPV OR human papillomavirus)

Table S1. PRISMA 2020 checklist.

Section and topic	Item #	Checklist item	Location where item is reported
		**Title**	
Title	**1**	Identify the report as a systematic review.	1
		**Abstract**	
Abstract	**2**	See the PRISMA 2020 for Abstracts checklist (Table 2).	3
		**Introduction**	
Rationale	**3**	Describe the rationale for the review in the context of existing knowledge.	4
Objectives	**4**	Provide an explicit statement of the objective(s) or question(s) the review addresses.	4
		**Methods**	
Eligibility criteria	**5**	Specify the inclusion and exclusion criteria for the review and how studies were grouped for the syntheses.	5
Information sources	**6**	Specify all databases, registers, websites, organisations, reference lists and other sources searched or consulted to identify studies. Specify the date when each source was last searched or consulted.	5
Search strategy	**7**	Present the full search strategies for all databases, registers and websites, including any filters and limits used.	Supplemetary material
Selection process	**8**	Specify the methods used to decide whether a study met the inclusion criteria of the review, including how many reviewers screened each record and each report retrieved, whether they worked independently, and if applicable, details of automation tools used in the process.	5
Data collection process	**9**	Specify the methods used to collect data from reports, including how many reviewers collected data from each report, whether they worked independently, any processes for obtaining or confirming data from study investigators, and if applicable, details of automation tools used in the process.	5
Data items	**10a**	List and define all outcomes for which data were sought. Specify whether all results that were compatible with each outcome domain in each study were sought (e.g. for all measures, time points, analyses), and if not, the methods used to decide which results to collect.	5
	**10b**	List and define all other variables for which data were sought (e.g. participant and intervention characteristics, funding sources). Describe any assumptions made about any missing or unclear information.	5
Study risk of bias assessment	**11**	Specify the methods used to assess risk of bias in the included studies, including details of the tool(s) used, how many reviewers assessed each study and whether they worked independently, and if applicable, details of automation tools used in the process.	6
Effect measures	**12**	Specify for each outcome the effect measure(s) (e.g. risk ratio, mean difference) used in the synthesis or presentation of results.	6
Synthesis methods	**13a**	Describe the processes used to decide which studies were eligible for each synthesis (e.g. tabulating the study intervention characteristics and comparing against the planned groups for each synthesis (item #5)).	6
	**13b**	Describe any methods required to prepare the data for presentation or synthesis, such as handling of missing summary statistics, or data conversions.	6
	**13c**	Describe any methods used to tabulate or visually display results of individual studies and syntheses.	6
	**13d**	Describe any methods used to synthesise results and provide a rationale for the choice(s). If meta-analysis was performed, describe the model(s), method(s) to identify the presence and extent of statistical heterogeneity, and software package(s) used.	6
	**13e**	Describe any methods used to explore possible causes of heterogeneity among study results (e.g. subgroup analysis, meta-regression).	6
	**13f**	Describe any sensitivity analyses conducted to assess robustness of the synthesised results.	6
Reporting bias assessment	**14**	Describe any methods used to assess risk of bias due to missing results in a synthesis (arising from reporting biases).	6
Certainty assessment	**15**	Describe any methods used to assess certainty (or confidence) in the body of evidence for an outcome.	6

		**Results**	
Study selection	**16a**	Describe the results of the search and selection process, from the number of records identified in the search to the number of studies included in the review, ideally using a flow diagram.	Figure 1
	**16b**	Cite studies that might appear to meet the inclusion criteria, but which were excluded, and explain why they were excluded.	Figure 1
Study characteristics	**17**	Cite each included study and present its characteristics.	Table 1
Risk of bias in studies	**18**	Present assessments of risk of bias for each included study.	8
Results of individual studies	**19**	For all outcomes, present, for each study: (a) summary statistics for each group (where appropriate) and (b) an effect estimate and its precision (e.g. confidence/credible interval), ideally using structured tables or plots.	8
Results of syntheses	**20a**	For each synthesis, briefly summarise the characteristics and risk of bias among contributing studies.	8
	**20b**	Present results of all statistical syntheses conducted. If meta-analysis was done, present for each the summary estimate and its precision (e.g. confidence/credible interval) and measures of statistical heterogeneity. If comparing groups, describe the direction of the effect.	8
	**20c**	Present results of all investigations of possible causes of heterogeneity among study results.	8
	**20d**	Present results of all sensitivity analyses conducted to assess the robustness of the synthesised results.	8
Reporting biases	**21**	Present assessments of risk of bias due to missing results (arising from reporting biases) for each synthesis assessed.	
Certainty of evidence	**22**	Present assessments of certainty (or confidence) in the body of evidence for each outcome assessed.	Tables S4 and S5
		**Discussion**	
Discussion	**23a**	Provide a general interpretation of the results in the context of other evidence.	10
	**23b**	Discuss any limitations of the evidence included in the review.	10
	**23c**	Discuss any limitations of the review processes used.	10
	**23d**	Discuss implications of the results for practice, policy, and future research.	11
		**Other information**	
Registration and protocol	**24a**	Provide registration information for the review, including register name and registration number, or state that the review was not registered.	5
	**24b**	Indicate where the review protocol can be accessed, or state that a protocol was not prepared.	5
	**24c**	Describe and explain any amendments to information provided at registration or in the protocol.	5
Support	**25**	Describe sources of financial or non-financial support for the review, and the role of the funders or sponsors in the review.	2
Competing interests	**26**	Declare any competing interests of review authors.	2
Availability of data, code, and other materials	**27**	Report which of the following are publicly available and where they can be found: template data collection forms; data extracted from included studies; data used for all analyses; analytic code; any other materials used in the review.	

Table S2. Imiquimod administration.

Study	dose&form	application	schedule	**cumulative dose**
Polerauer et al [1]	6.25 mg Imiquimod suppository	by patient	**16 weeks** (1-2 w:1x, 3-4w: 2x, 5-16w: 3x),	**243.75 mg/patient**
Grimm et al [2]	6.25 mg Imiquimod suppository	by patient	**16 weeks** (1-2 w:1x, 3-4w: 2x, 5-16w: 3x)	**243.75 mg/patient**
Fonseca et al [3]	250 mg of 5% Imiquimod cream (12,5 mg active substance)	by doctor	**12 weeks** (1x a week)	**150 mg/patient**
Hendriks et al [4]	6.25 mg Imiquimod cream	by patient	**16 weeks** (3x a week)	**300 mg/patient**
Kim et al [5]	12.5 mg Imiquimod cream	by doctor	**8 weeks** (1x a week -median value)	**100 mg/patient**
Cokan et al [6]	250 mg of 5% Imiquimod cream	by patient	**16 weeks** (3x a week)	**600 mg/patient**
Lin et al [7]	250 mg of 5% Imiquimod cream	by patient	minimum **12 doses**	**150 mg/ patient**
Pachmann et al [8]	50 mg of 5 % imiquimod cream (2,5 mg active substance)	by doctor	**5 times** applied by doctor	**12.5 mg/patient**

Table S3. Grade for outcomes that assessed Imiquimod compared to conization.

Certainty assessment							№ of patients		Effect		Certainty	Importance
№ of studies	Study design	Risk of bias	Incon-sistency	Indirect-ness	Impreci-sion	Other considera-tions	Imiquimod	Conization	Relative (95% CI)	Absolute (95% CI)		
Imiquimod compared to conization in the ITT analysis												
4	observa-tional studies	not serious	not serious	not serious	not serious	publication bias strongly suspected[a]	109/209 (52.2%)	169/196 (86.2%)	**RR 0.62** (0.42 to 0.92)	**328 fewer per 1 000** (from 500 fewer to 69 fewer)	⊕⊕⊕◯ Moderate	
Imiquimod compared to conization in the PP analysis												
3	random-ised trials	not serious	not serious	not serious	not serious	publication bias strongly suspected[a]	82/127 (64.6%)	111/134 (82.8%)	**RR 0.78** (0.56 to 1.07)	**182 fewer per 1 000** (from 364 fewer to 58 more)	⊕⊕⊕◯ Moderate	

CI: confidence interval; **RR:** risk ratio. [a] funnel plot show's asymmetry.

Table S4. Grade for outcomes that assessed Imiquimod compared to control on HPV clearance.

Certainty assessment							№ of patients		Effect		Certainty	Importance
№ of studies	Study design	Risk of bias	Incon-sistency	Indirect-ness	Impreci-sion	Other considera-tions	Imiquimod	control	Relative (95% CI)	Absolute (95% CI)		
Imiquimod compared to control on HPV clearance in the ITT analysis												
5	observa-tional studies	serious[a]	serious[b]	serious[c]	serious[d]	none	100/196 (51.0%)	86/180 (47.8%)	**RR 1.29** (0.52 to 3.21)	**139 more per 1 000** (from 229 fewer to 1 000 more)	⊕◯◯◯ Very low	
Imiquimod compared to control on HPV clearance in the PP analysis												
3	random-ised trials	not serious	serious[b]	serious[c]	serious[d]	none	59/100 (59.0%)	47/81 (58.0%)	**RR 1.27** (0.10 to 16.29)	**157 more per 1 000** (from 522 fewer to 1 000 more)	⊕◯◯◯ Very low	

CI: confidence interval; **RR:** risk ratio. [a] Lin et al high risk of bias as they use a historical control group. [b] Large variation in effect, and confidence interval don't overlap in few studies. [c] Important differences in population. [d] wide confidence interval.

Table S5. Grade on HPV 16/18 clearance compared to other HR-HPV clearance.

Certainty assessment							№ of patients		Effect		Certainty	Importance
№ of studies	Study design	Risk of bias	Incon-sistency	Indirect-ness	Impreci-sion	Other considera-tions	Imiquimod	control	Relative (95% CI)	Absolute (95% CI)		
4	observa-tional studies	not serious	not serious	not serious	serious[e]	none	40/80 (50.0%)	48/88 (54.5%)	**RR 0.89** (0.58 to 1.37)	**60 fewer per 1 000** (from 229 fewer to 202 more)	⊕◯◯◯ Very low	

CI: confidence interval; **RR:** risk ratio. [e] Small number of events.

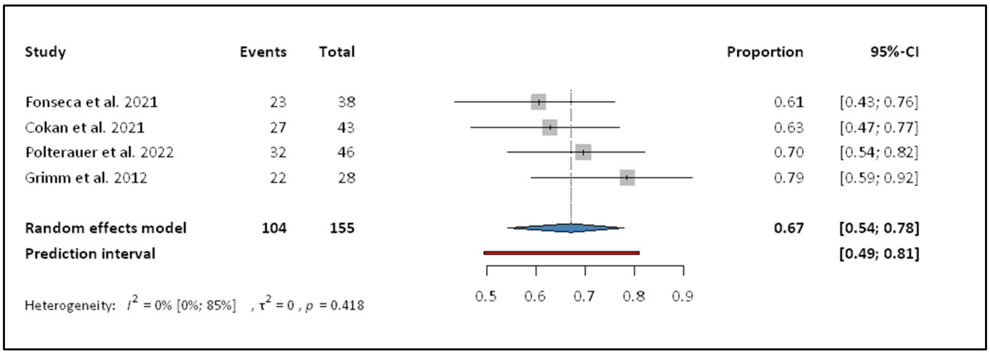

Figure S1. Forest plot of studies representing Imiquimod and CIN 2-3 regression in the PP analysis.

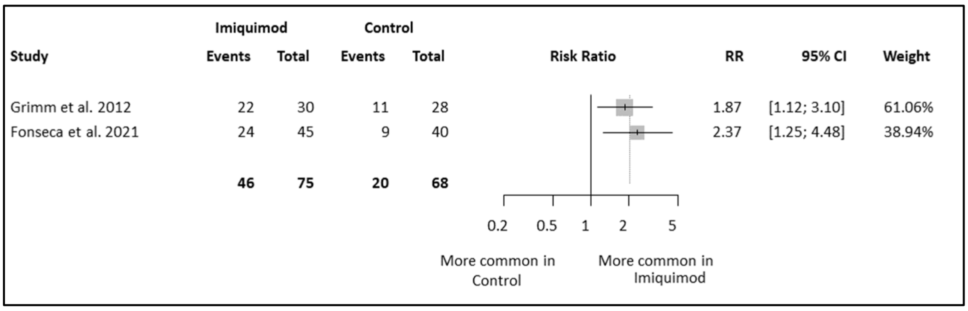

Figure S2. Forest plot of studies representing Imiquimod compared to no intervention on CIN 2-3 regression.

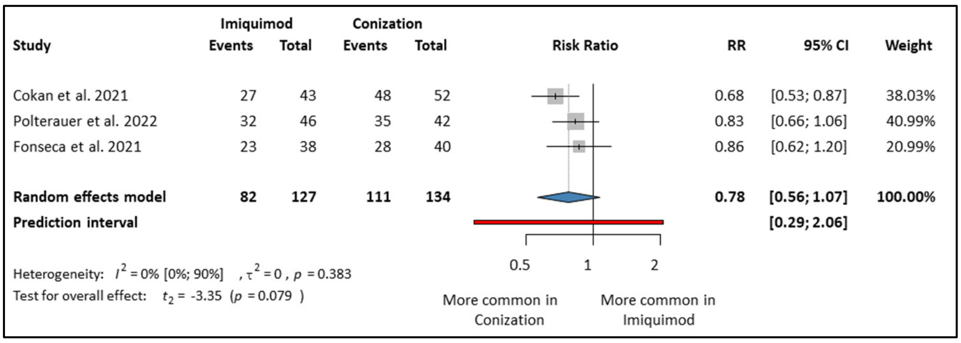

Figure S3. Forest plot of studies representing Imiquimod group compared to conization on CIN 2-3 regression in the PP analysis.

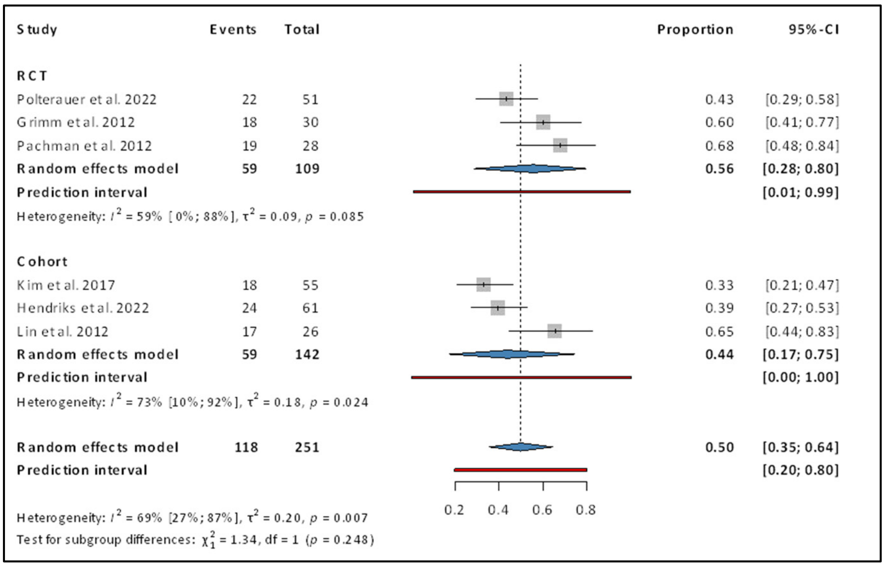

Figure S4. Forest plot of studies representing Imiquimod on HPV clearance according to study type.

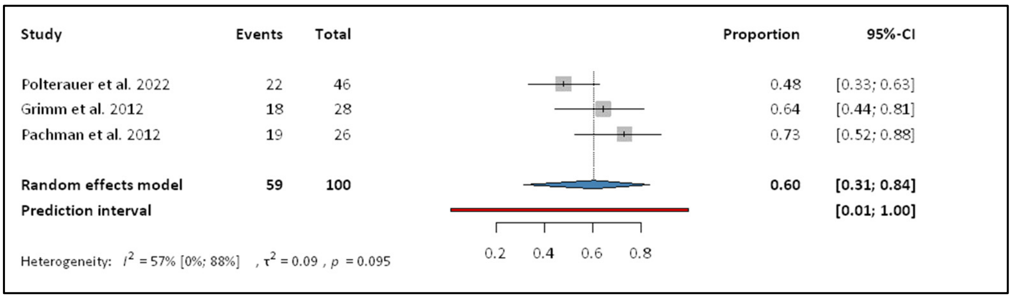

Figure S5. Forest plot of studies representing Imiquimod on HPV clearance in the PP analysis.

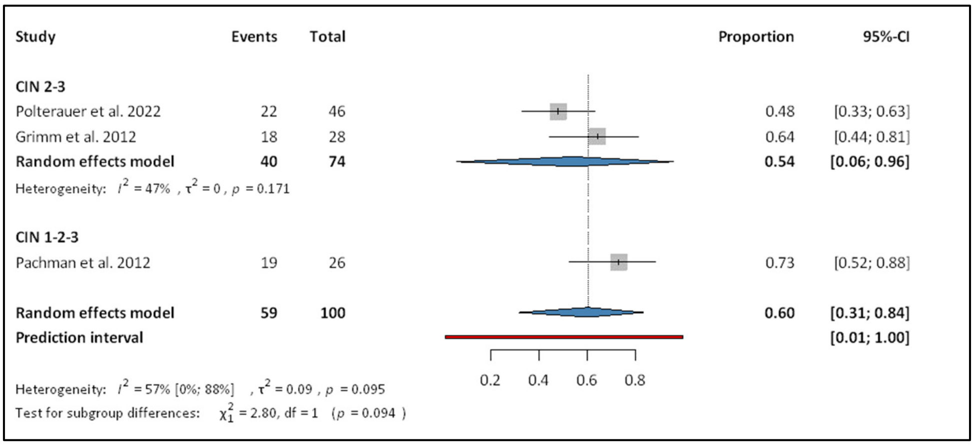

Figure S6. Forest plot of studies representing Imiquimod on HPV clearance according to subgroup analysis of CIN status in the PP analysis.

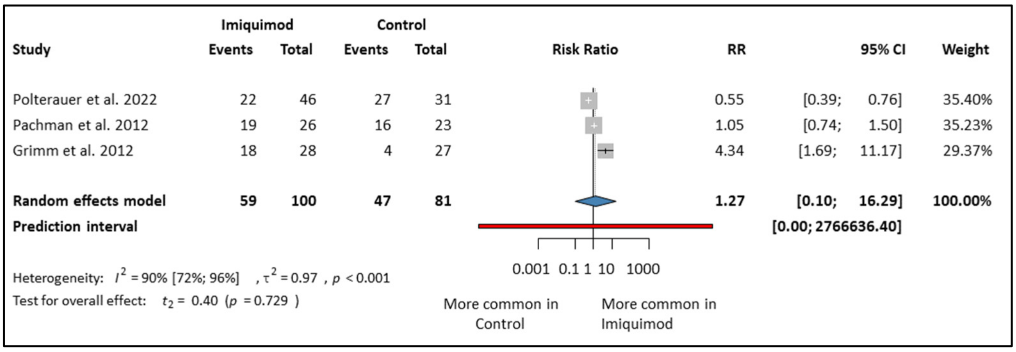

Figure S7. Forest plot of studies representing the Imiquimod group compared to control on HPV clearance in the PP analysis.

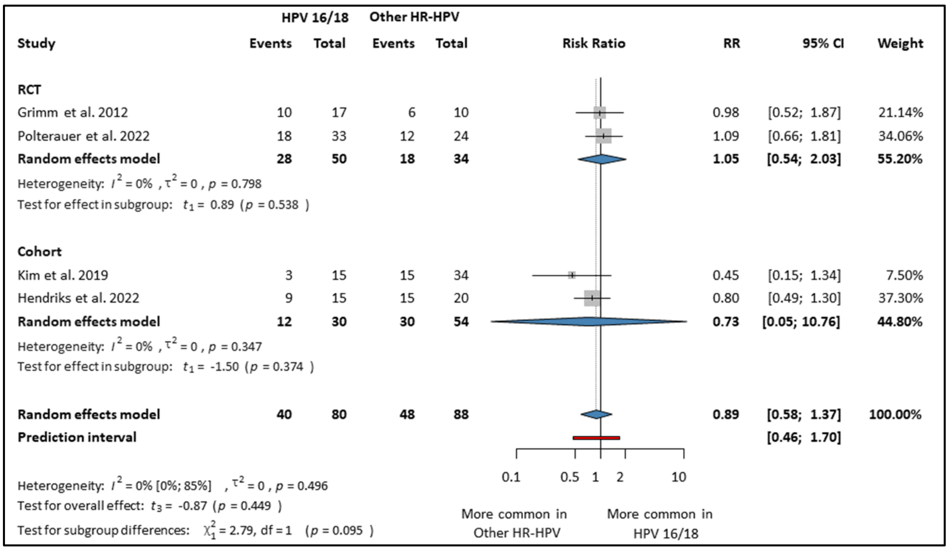

Figure S8. Forest plot of studies representing the HPV 16/18 clearance compared to other HR-HPV clearance in the Imiquimod group.

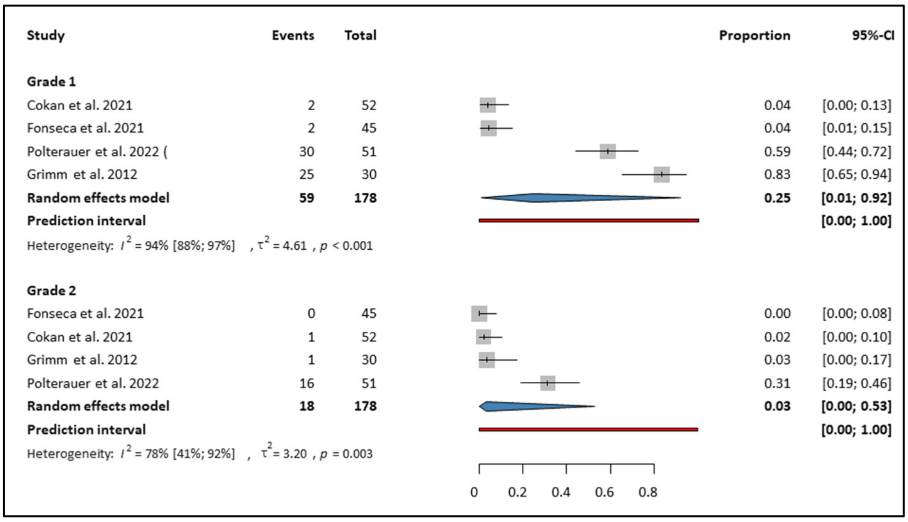

Figure S9. Forest plot of studies representing the occurrence of headaches in patients treated with Imiquimod.

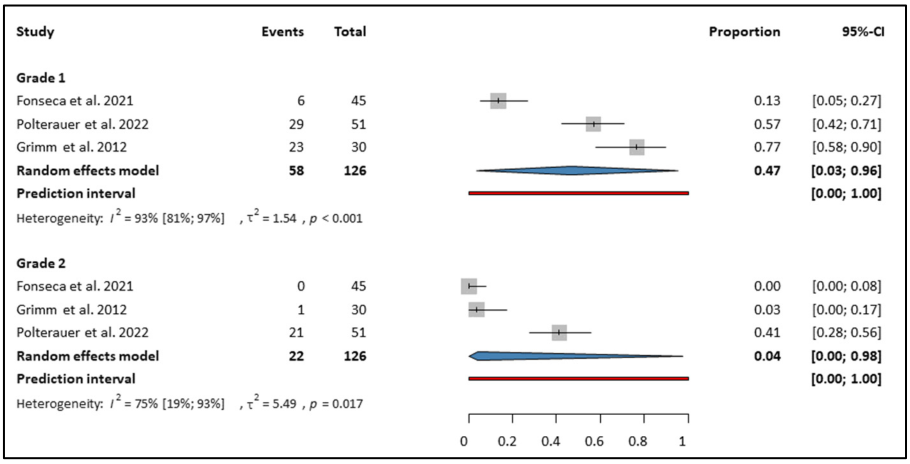

Figure S10. Forest plot of studies representing the occurrence of myalgia in patients treated with Imiquimod.

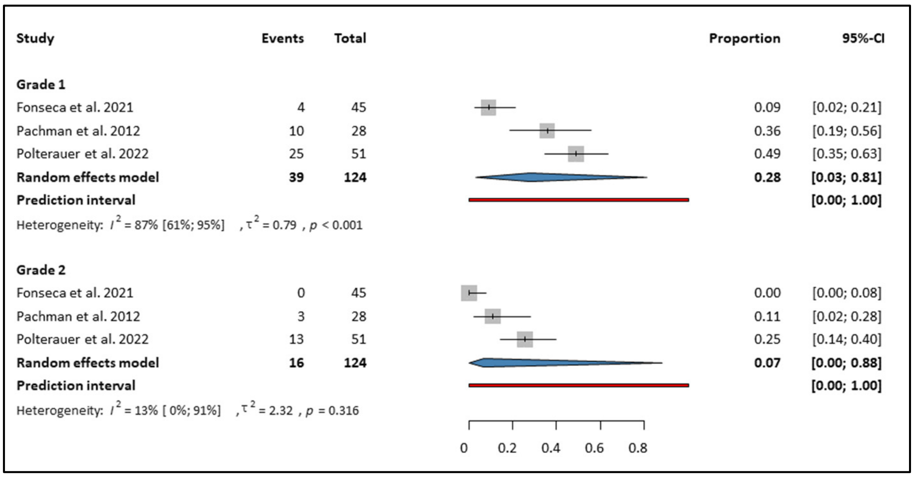

Figure S11. Forest plot of studies representing the occurrence of fatigue in patients treated with Imiquimod.

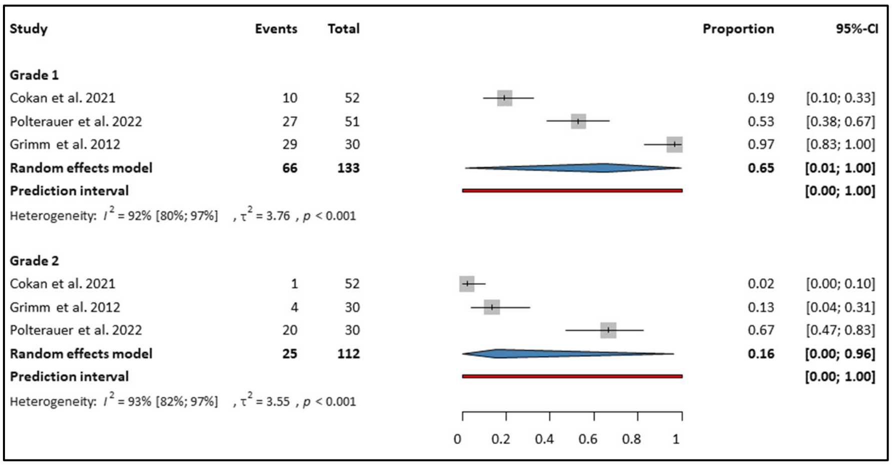

Figure S12. Forest plot of studies representing the occurrence of flu-like symptoms in patients treated with Imiquimod.

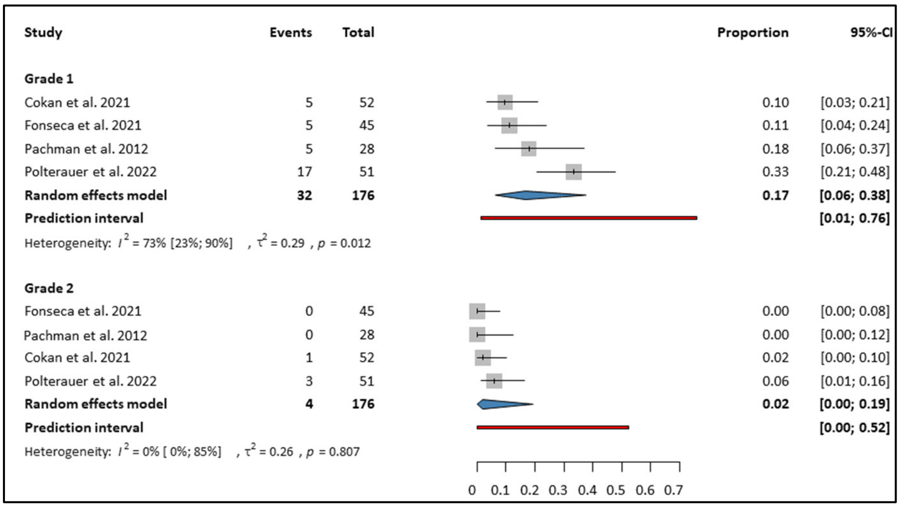

Figure S13. Forest plot of studies representing the occurrence of fever in patients treated with Imiquimod.

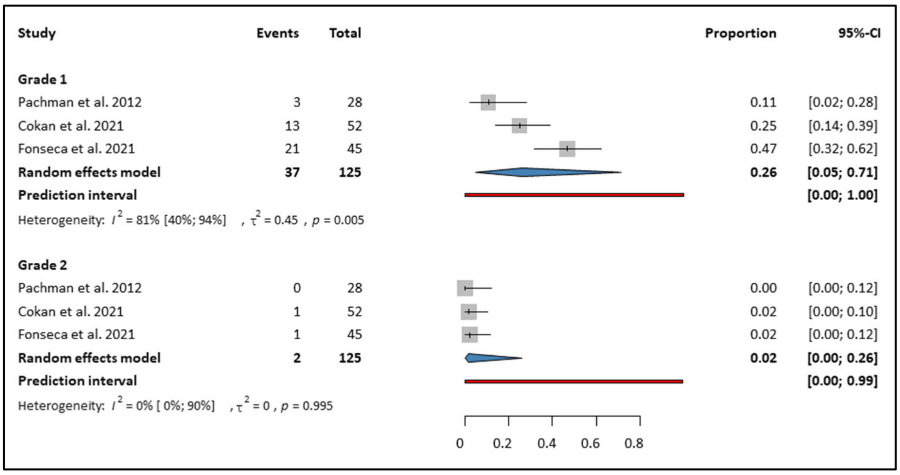

Figure S14. Forest plot of studies representing the occurrence of abdominal pain in patients treated with Imiquimod.

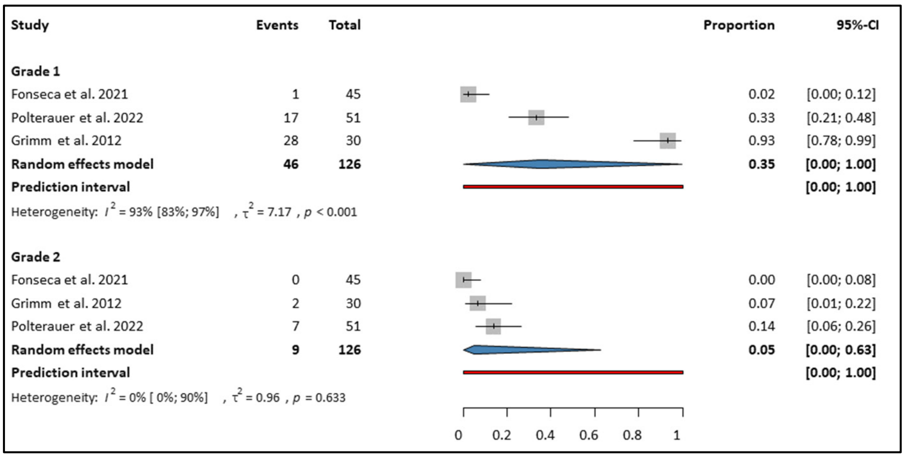

Figure S15. Forest plot of studies representing the occurrence of vaginal pruritus in patients treated with Imiquimod.

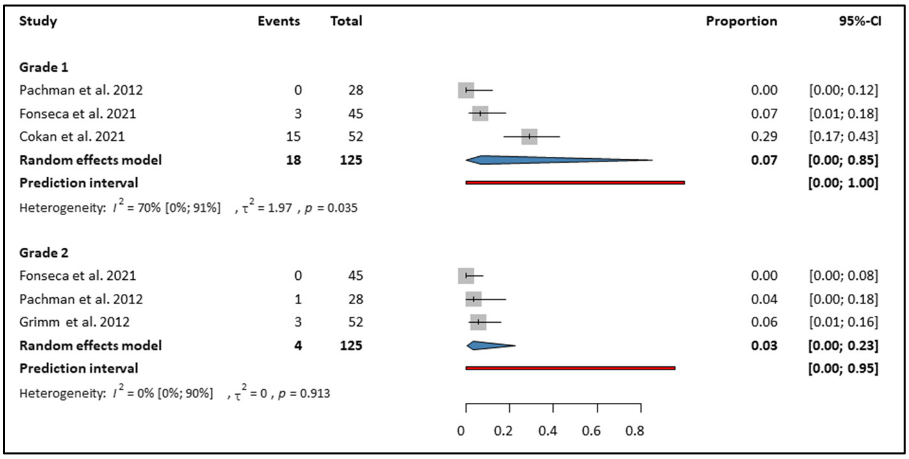

Figure S16. Forest plot of studies representing the occurrence of vaginal bleeding in patients treated with Imiquimod.

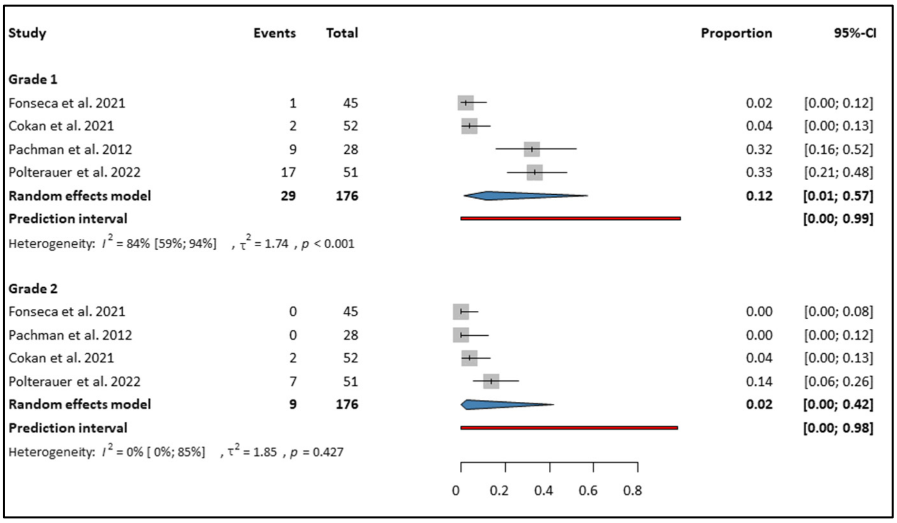

Figure S17. Forest plot of studies representing the occurrence of vaginal discharge in patients treated with Imiquimod.

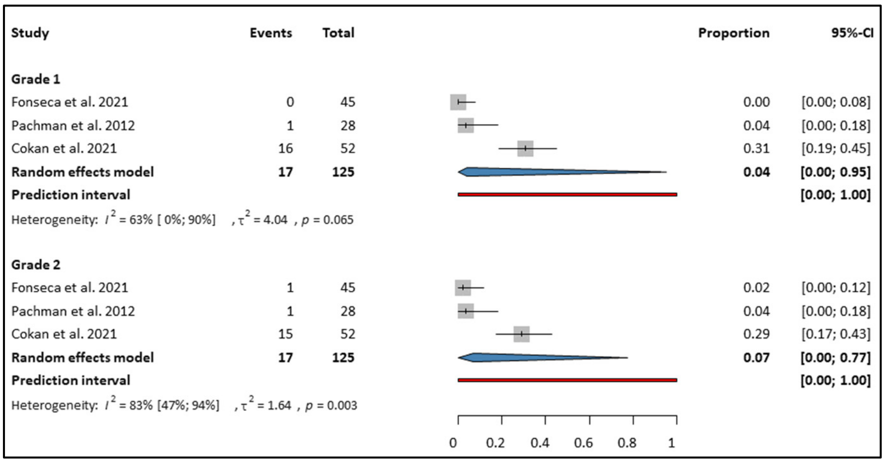

Figure S18. Forest plot of studies representing the occurrence of inflammation of the vagina in patients treated with Imiquimod.

Study	D1	D2	D3	D4	D5	Overall
Polterauer et al. 2022	Low	Some concerns	Low	Low	Low	Some concerns
Fonseca et al. 2021	Low	Some concerns	Low	Low	Low	Some concerns
Cokan et al. 2021	Low	Some concerns	Some concerns	Low	Low	Some concerns

Risk of bias 2. assessment of studies investigated **Imiquimod** compared to **conization in the ITT group**

Study	D1	D2	D3	D4	D5	Overall
Polterauer et al. 2022	Low	Some concerns	Low	Low	Low	Some concerns
Fonseca et al. 2021	Low	Some concerns	Low	Low	Low	Some concerns
Cokan et al. 2021	Low	Some concerns	Some concerns	Low	Low	Some concerns

Risk of bias 2. assessment of studies investigated **Imiquimod** compared to **conization in the PP group**

Study	D1	D2	D3	D4	D5	Overall
Grimm et al. 2012	Low	Low	Low	Low	Low	Low
Polterauer et al. 2022	Some concerns	Low	Low	Low	Low	Some concerns
Pachman et al. 2012	Some concerns	Low	Some concerns	Low	Low	Some concerns

Risk of bias 2. assessment of studies investigated **Imiquimod** compared to control (**HPV clearance) in the ITT group**

Study	D1	D2	D3	D4	D5	Overall
Grimm et al. 2012	Low	Some concerns	Low	Low	Low	Low
Polterauer et al. 2022	Low	Some concerns	Low	Low	Low	Some concerns
Pachman et al. 2012	Some concerns	Low	Some concerns	Low	Low	Some concerns

Risk of bias 2. assessment of studies investigated **Imiquimod** compared to control (**HPV clearance) in the PP group**

Study	D1	D2	D3	D4	D5	Overall
Grimm et al. 2012	Low	Some concerns	Low	Low	Low	Low
Polterauer et al. 2022	Low	Some concerns	Low	Low	Low	Some concerns

Risk of bias assessment 2. of studies investigated **HPV 16/18 clearance and other HR-HPV clearance**

Figure S19. Risk of bias assessment of randomized control studies. D1: Risk of bias arising from the randomization process, D2: Risk of bias due to deviations from the intended interventions, D4: Risk of bias due to missing outcome data, D4: Risk of bias in measurement of the outcome, D5: Risk of bias in selection of the reported result.

Study	D1	D2	D3	D4	D5	D6	D7	Overall
Hendiks et al. 2022	Low	Low	Low	Moderate	Low	Low	Low	Moderate

Robinson I. assessment of studies investigated **Imiquimod** compared to **conization in the ITT group**

Study	D1	D2	D3	D4	D5	D6	D7	Overall
Hendriks et al. 2022	Low	Low	Low	Moderate	Low	Low	Low	Moderate
Lin et al. 2012	Serious	Moderate	Moderate	Low	Low	Low	Moderate	Serious

Robinson I. assessment of studies investigated **Imiquimod** compared to control **(HPV clearance**) in the **ITT group**

Study	D1	D2	D3	D4	D5	D6	D7	Overall
Hendriks et al. 2022	Low	Low	Low	Moderate	Low	Low	Low	Moderate
Kim et al. 2019	Low	Moderate	Moderate	Low	Low	Low	Moderate	Moderate

Robinson I. assessment studies investigated **HPV 16/18 clearance and other HR-HPV clearance**

Figure S20. Risk of bias assessment of non-randomized controlled studies. D1: Bias due to confounding, D2: Bias in selection of participants into the study, D3: Bias in classification of interventions, D4: Bias due to deviation from intended intervention, D5: Bias due to missing data, D6: Bias in measurement of outcomes, D7 Bias in selected results.

Study	1	2	3	4	5	6	7	8	9
Grimm et al. 2012	Yes	Yes	Yes	Yes	Yes	Yes	Yes	Yes	Yes
Polterauer et al. 2022	Yes	Yes	No	No	Yes	Yes	Yes	Yes	Yes
Fonseca et al. 2021	Yes	Yes	Yes	Yes	Yes	Yes	Yes	Yes	Yes
Hendriks et al.2022	Yes	Yes	Yes	Yes	Yes	Yes	Yes	Yes	Unclear
Kim et al. 2019	Yes	Unclear	No	No	Yes	Yes	Yes	No	Yes
Cokan et al. 2021	Yes	Yes	Yes	No	Yes	Yes	Yes	Yes	Yes

JBI Critical Appraisal Checlist of studies that investigated **Imiquimod** and **CIN regression in the ITT group**

Study	1	2	3	4	5	6	7	8	9
Grimm et al. 2012	Yes	Yes	Yes	Yes	Yes	Yes	Yes	Yes	Yes
Polterauer et al. 2022	Yes	Yes	No	No	Yes	Yes	Yes	Yes	Yes
Fonseca et al. 2021	Yes	Yes	Yes	Yes	Yes	Yes	Yes	Yes	Yes
Cokan et al. 2021	Yes	Yes	Yes	No	Yes	Yes	Yes	Yes	Yes

JBI Critical Appraisal Checlist of studies that investigated **Imiquimod** and **CIN regression in the PP group**

Study	1	2	3	4	5	6	7	8	9
Grimm et al. 2012	Yes	Yes	Yes	Yes	Yes	Yes	Yes	Yes	Yes
Polterauer et al. 2022	Yes	Yes	No	No	Yes	Yes	Yes	Yes	Yes
Hendriks et al. 2022	Yes	Yes	Yes	Yes	Yes	Yes	Yes	Yes	Unclear
Pachman et al. 2012	Yes	Yes	No	Yes	Unclear	Yes	Yes	Yes	Yes
Lin et al. 2012	Yes	Unclear	No	No	No	Yes	Yes	No	Yes
Kim et al. 2019	Yes	Unclear	No	No	Yes	Yes	Yes	No	Yes

JBI Critical Appraisal Checlist of studies that investigated **Imiquimod** and **HPV clearance in the ITT group**

Study	1	2	3	4	5	6	7	8	9
Grimm et al. 2012	Yes	Yes	Yes	Yes	Yes	Yes	Yes	Yes	Yes
Polterauer et al. 2022	Yes	Yes	No	No	Yes	Yes	Yes	Yes	Yes
Pachman et al. 2012	Yes	Yes	No	Yes	Unclear	Yes	Yes	Yes	Yes

JBI Critical Appraisal Checlist of studies that investigated **Imiquimod** and **HPV clearance in the PP group**

Figure S21. JBI Critical Appraisal Checklist for response rate outcomes. 1: Was the sample frame appropriate to address the target population?, 2: Were the study participants sampled in an appropriate way?, 3: Was the sample size adequate? 4: Were the study subjects and the setting described in detail?, 5: Was the data analysis conducted with sufficient coverage of the identified sample?, 6: Were valid methods used for the identification of the condition?, 7: Was the condition measured in a standard, reliable way for all participants?, 8, Was there appropriate statistical analysis?, 9: Was the response rate adequate, and if not, was the low response rate managed appropriately?

References

1. Polterauer, S.; Reich, O.; Widschwendter, A.; Hadjari, L.; Bogner, G.; Reinthaller, A.; Joura, E.; Trutnovsky, G.; Ciresa-Koenig, A.; Ganhoer-Schimboeck, J.; et al., Topical imiquimod compared with conization to treat cervical high-grade squamous intraepithelial lesions: multicenter, randomized controlled trial. *Gynecologic oncology* **2022,** *165* (1), 23-29.
2. Grimm, C.; Polterauer, S.; Natter, C.; Rahhal, J.; Hefler, L.; Tempfer, C. B.; Heinze, G.; Stary, G.; Reinthaller, A.; Speiser, P., Treatment of cervical intraepithelial neoplasia with topical imiquimod: a randomized controlled trial. *Obstetrics and gynecology* **2012,** *120* (1), 152-159.
3. Fonseca, B. O.; Possati-Resende, J. C.; Salcedo, M. P.; Schmeler, K. M.; Accorsi, G. S.; Fregnani, J. H. T. G.; Antoniazzi, M.; Pantano, N. P.; Santana, I. V. V.; Matsushita, G. M.; Dos Reis, R., Topical Imiquimod for the Treatment of High-Grade Squamous Intraepithelial Lesions of the Cervix: A Randomized Controlled Trial. *Obstetrics and gynecology* **2021,** *137* (6), 1043-1053.
4. Hendriks, N.; Koeneman, M. M.; Van De Sande, A. J. M.; Penders, C. G. J.; Piek, J. M. J.; Kooreman, L. F. S.; Van Kuijk, S. M. J.; Hoosemans, L.; Sep, S. J. S.; De Vos Van Steenwijk, P. J.; Van Beekhuizen, H. J.; Slangen, B. F. M.; Nijman, H. W.; Kruitwagen, R. F. P. M.; Kruse, A. J., Topical Imiquimod Treatment of High-grade Cervical Intraepithelial Neoplasia (TOPIC-3): A Nonrandomized Multicenter Study. *Journal of Immunotherapy* **2022,** *45* (3), 180-186.
5. Kim, J. H.; Kim, D. Y., Imiquimod as an alternative option for young women with high-grade cervical intraepithelial neoplasia. *European Journal of Gynaecological Oncology* **2019,** *40* (6), 943-947.
6. Cokan, A.; Pakiž, M.; Serdinšek, T.; Dovnik, A.; Kodrič, T.; Fokter, A. R.; Kavalar, R.; But, I., Comparison of conservative treatment of cervical intraepithelial lesions with imiquimod with standard excisional technique using lletz: A randomized controlled trial. *Journal of Clinical Medicine* **2021,** *10* (24).
7. Lin, C. T.; Qiu, J. T.; Wang, C. J.; Chang, S. D.; Tang, Y. H.; Wu, P. J.; Jung, S. M.; Huang, C. C.; Chou, H. H.; Jao, M. S.; Lai, C. H., Topical imiquimod treatment for human papillomavirus infection in patients with and without cervical/vaginal intraepithelial neoplasia. *Taiwanese Journal of Obstetrics and Gynecology* **2012,** *51* (4), 533-538.
8. Pachman, D. R.; Barton, D. L.; Clayton, A. C.; McGovern, R. M.; Jefferies, J. A.; Novotny, P. J.; Sloan, J. A.; Loprinzi, C. L.; Gostout, B. S., Randomized clinical trial of imiquimod: An adjunct to treating cervical dysplasia. *American Journal of Obstetrics and Gynecology* **2012,** *206* (1), 42.e1-42.e7.
